# Draft Genome Sequence of a 94-Year-Old *Listeria monocytogenes* Isolate, SLCC208

**DOI:** 10.1128/genomeA.01572-15

**Published:** 2016-01-21

**Authors:** Patrick Hyden, Ariane Pietzka, Franz Allerberger, Burkhard Springer, Christoph Sensen, Werner Ruppitsch

**Affiliations:** a Institute of Molecular Biotechnology, Graz University of Technology, Graz, Austria; b Institute of Medical Microbiology and Hygiene, Austrian Agency for Health and Food Safety, Graz, Austria; c Department of Biotechnology, University of Natural Resources and Life Sciences, Vienna, Austria

## Abstract

We report here the draft genome sequence of *Listeria monocytogenes* strain SLCC208 from Seeliger’s historical Special *Listeria* Culture Collection, initially cultured from a human case in France in 1921. This is, to our knowledge, the oldest *L. monocytogenes* isolate available and may be useful for comparative genomic studies of *L. monocytogenes*.

## GENOME ANNOUNCEMENT

*Listeria monocytogenes*, the causative agent of listeriosis, is a facultative intracellular, facultative anaerobic, Gram-positive, psychrophilic, and salt-tolerant pathogen of humans and animals. *L. monocytogenes* is spread widely in the environment. This bacterial species has the ability to survive and grow under extreme conditions (1). The characteristic symptoms of listeriosis comprise encephalitis, meningitis, gastroenteritis, and septicemia. With a high case-fatality rate of up to 30%, *L. monocytogenes* is a considerable human pathogen (2). Nearly all cases of listeriosis are caused by the consumption or use of contaminated food or feed. In listeriosis outbreaks and for epidemiological investigations, subtyping of *L. monocytogenes* is essential (3). *L. monocytogenes* strains can be categorized by serotyping into 12 serotypes (4), of which 4b, 1/2a, and 1/2 b isolates cause about 96% of all reported human listeriosis cases (5).

*L. monocytogenes* isolate SLCC208 was subcultured from the original agar slant of the Seeliger collection by adding Trypticase soy broth (bioMérieux, Marcy I’Etoile, France) and subculturing on Columbia blood agar plates (bioMérieux). After another subculture step using RAPID’L.Mono plates (Bio-Rad, Vienna, Austria) for species verification, overnight cultures grown on Columbia blood agar plates (bioMérieux) were used for isolation of genomic DNA using the MagAttract high-molecular-weight (HMW) DNA kit, according to the instructions of the manufacturer (Qiagen, Hilden, Germany). Serotype PCR was performed (6) for confirmation of the determined serotype 4b of isolate SLCC208. The fragment library was constructed with the Nextera XT kit, as recommended by the manufacturer (Illumina, Inc., San Diego, CA, USA), and 2 × 300-bp fragments were sequenced on a MiSeq (Illumina, Inc.), generating 1,121,712 reads from 281,542,985 unassembled nucleotides. Raw reads were preprocessed using Trimmomatic 0.32 (7) for adapter trimming, as recommended by the authors, and quality trimming for average Q30 in a window of 20 bp. The preprocessed reads were *de-novo* assembled into a draft genome using SPAdes version 3.5.0 (8). Contigs were filtered for a minimum coverage of 5× and minimum length of 200 bp, which resulted in 22 contigs with a total of 2,916,998 nucleotides at 56-fold coverage. A total of 2,930 genes, 2,842 coding sequences, 19 pseudogenes, 11 rRNA operons (6 complete and 5 partial), and 58 tRNA genes were identified by the NCBI Prokaryotic Genome Automatic Annotation Pipeline.

Extraction of the classical multilocus sequence type (MLST) from the draft genome assigned strain SLCC208 to sequence type 495 (ST495) and clonal complex 1 (CC1) (9).

### Nucleotide sequence accession numbers.

This whole-genome shotgun project has been deposited at DDBJ/ENA/GenBank under the accession number LMXJ00000000. The version described in this paper is the first version, LMXJ01000000.
